# A Rare Case of Extremely Severe Heterotopic Ossification after Primary Total Hip Arthroplasty due to Persistent Mild Periprosthetic Joint Infection

**DOI:** 10.1155/2021/8849929

**Published:** 2021-05-22

**Authors:** Yutaka Kinoshita, Shunji Nakano, Shinji Yoshioka, Masaru Nakamura, Tomohiro Goto, Daisuke Hamada, Koichi Sairyo

**Affiliations:** ^1^Department of Orthopedics, Tokushima Municipal Hospital, Tokushima, Japan; ^2^Department of Orthopedics, Institute of Health Biosciences, Tokushima University Graduate School, Tokushima, Japan

## Abstract

Periprosthetic joint infection (PJI) caused by coagulase-negative staphylococci (CNS) can be a mild, persisting infection. Although heterotopic ossification (HO) is common following total hip arthroplasty (THA), the etiology of severe HO remains unclear. Herein, we describe a rare case of extremely severe HO after a PJI associated with a *Staphylococcus caprae* infection in a 78-year-old male patient. The patient had poorly controlled diabetes mellitus with no diabetic complications. The patient had no previous history of hip surgery, hip injury, or systemic bacterial infection. Immediately after the initial THA, he developed intermittent low-grade fever (37°C), which persisted for 3 months; consequently, he also reported mild hip pain during walking. He experienced a gradual decrease in hip range of motion within 5 years after the surgery, with progressive gait impairment. Two revision surgeries were required for the successful treatment of this difficult case. The patient's hip function improved, and the PJI was controlled following the second revision surgery. Based on the clinical course, CNS-caused PJI may lead to severe HO. This possibility warrants verification from an accumulated number of cases.

## 1. Introduction

Heterotopic ossification (HO) is characterized by the abnormal presence of bone in soft tissues. It has an incidence of 0.6%-90% [1] and is common after total hip arthroplasty (THA), with an incidence of 30%-40% after THA [2]. Although HO after THA is generally asymptomatic, severe HO can lead to postoperative hip pain, restricted hip range of motion (ROM), and bony impingement, resulting in THA instability [3]. The known risk factors for HO after THA include male sex, advanced age, previous HO, hypertrophic osteoarthritis, preoperative hip ankylosis, cemented prosthesis or previous surgery using the anterolateral or direct lateral approaches, and postoperative infection [3–9]. However, the specific etiology of HO, particularly that of severe HO, remains uncertain.

Previous reports suggested infection as a possible cause of HO [4]. Particularly, infection caused by the coagulase-negative staphylococcus (CNS) strain—*Staphylococcus caprae* (*S. caprae*)—was associated with periprosthetic joint infection (PJI) [10–14]. To our knowledge, only one report has described the development of severe HO associated with *S. caprae*-related PJI; however, whether a possible causal relationship existed between this infection and HO was not mentioned [15]. Herein, we describe a rare case of extremely severe HO after primary THA, which was associated with a persistent mild *S. caprae* infection and required two THA revisions due to acetabular cup loosening.

## 2. Case Presentation

The patient was a 78-year-old man who underwent cemented THA at another hospital to treat bilateral secondary osteoarthritis of the hip 17 years earlier. The surgeon had performed acetabuloplasty for hip dysplasia using an autologous femoral head-neck bone graft, and fixation was performed using metal screws. The patient's relevant medical history included poorly controlled diabetes mellitus (7.9% glycated hemoglobin), with no diabetic complications (i.e., sensory nerve dysfunction, gangrene, or superficial wounds). The patient had no history of hip surgery, hip injury, or systemic bacterial infection. Because the patient experienced no complications with respect to his surgical wounds, he did not undergo wound irrigation. Immediately after the initial THA, the patient developed intermittent low-grade fever (37°C), which persisted for 3 months, and was treated with antibiotics. No other details of this initial episode were available. Similarly, the patient reported mild hip pain during walking after the surgery. He experienced a gradual decrease in hip ROM within 5 years after the surgery, with progressive gait impairment.

**Figure 1:**
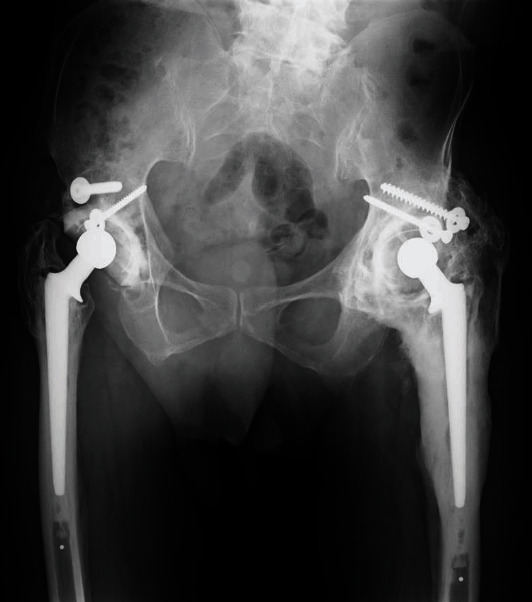
Plain radiograph obtained at the initial visit of a 78-year-old man, showing severe heterotopic ossification around the left hip joint.

**Figure 2:**
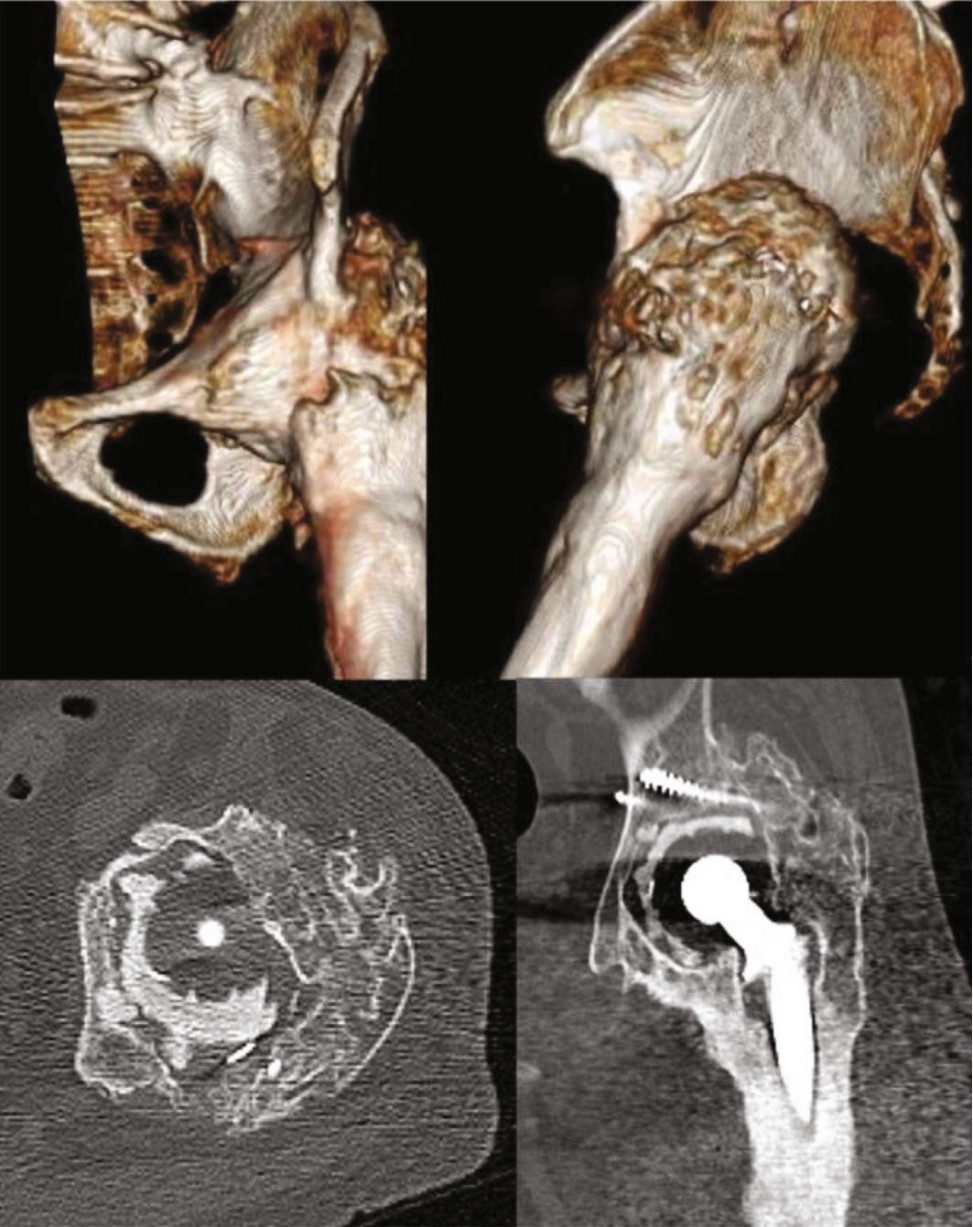
Three-dimensional computed tomography images showing severe heterotopic ossification around the hip joint and loosening of the acetabular cup in a 78-year-old man.

On initial examination at our hospital, plain radiographs revealed hip joint ankylosis, with evidence of severe HO (Brooker IV) around the hip joint and loosening of the acetabular cup (Figures 1 and 2). The patient had not received any prophylactic treatment for HO. Based on the patient's infection history, a chronic periprosthetic infection was suspected; however, preoperative aspiration could not be performed owing to hip joint ankylosis.

**Figure 3:**
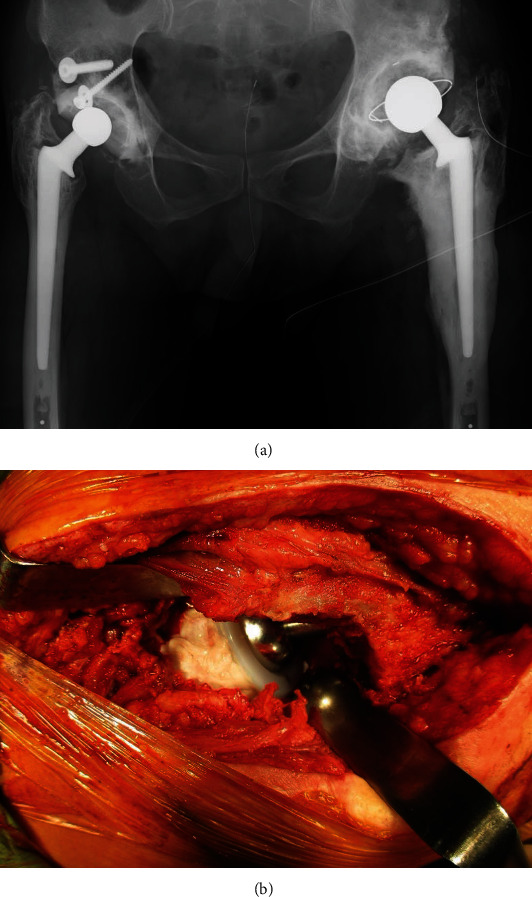
(a) Plain radiograph obtained immediately after the revision THA (second THA) in a 78-year-old man. Abbreviations: THA: total hip arthroplasty. (b) Owing to limited surgical field for a joint revision procedure in a 78-year-old man, using the standard approach because of hip ankylosis, the cement and polyethylene liner were placed onto the acetabulum after impaction of the new head.

**Figure 4:**
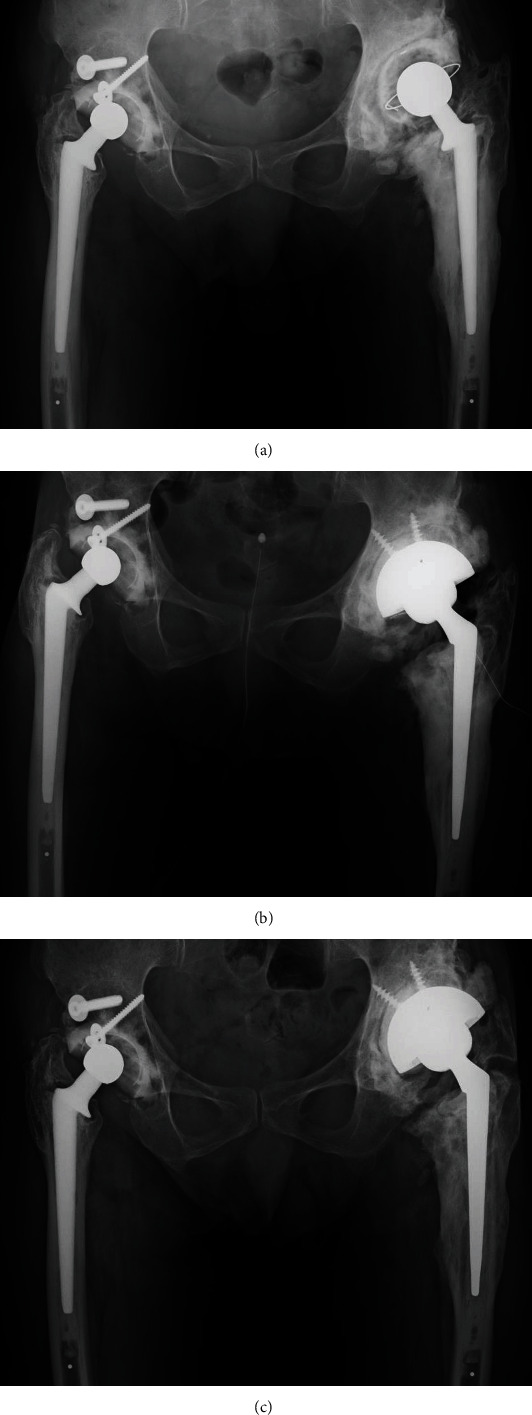
(a) Plain radiograph of a 78-year-old man obtained prior to the third THA, showing a clear zone around the cemented cup. Abbreviations: THA: total hip arthroplasty. (b) Plain radiograph of a 78-year-old man obtained immediately after the third THA, showing the initial gap between the host bone and the allograft in zones 2 and 3. We used vancomycin-mixed cement and replaced the cup and cemented stem simultaneously via a single-stage exchange. (c) Plain radiograph of a 78-year-old man obtained at the final follow-up (3 years postoperatively) examination with the gap between the host bone and allograft persisting in zone 3, with no evidence of prosthesis loosening.

We proceeded with revision THA (second THA) using a direct lateral approach that provided a wide anterior-to-posterior view of the surgical field. We first excised the HO and removed the loosened acetabular cup and fibrous tissue, followed by the impaction of bone grafting using an allograft. With no evidence of loosening of the femoral stem and considering the difficulty of performing THA in the presence of hip ankylosis, we did not replace the femoral stem (Figure 3(a)). As the working space was restricted by the hip ankylosis, we used a paradoxical approach for the revision THA. Specifically, we implanted a new metal femoral head, applied vancomycin-mixed cement to the acetabulum, and inserted and fixed a polyethylene liner into the narrow space between the metal head and the acetabulum on the unhardened cement (Figure 3(b)). There was no intraoperative evidence of pus or infectious debris. In addition, the patient's C-reactive protein (CRP) level and white blood cell count were low (0.6 mg/dL and 3600/*μ*L, respectively; normal CRP level: <0.3 mg/dL) at the initial visit to our hospital; thus, we refrained from using an antibiotic spacer or planning a two-stage approach. Although intraoperative Gram staining was negative, a bacterial culture of fluid from the hip joint revealed the presence of *S. caprae*. Intraoperative frozen sample analysis, which provides greater specificity than Gram staining, was not available in our hospital. We administered intravenous antibiotics, following our standard postoperative care guidelines for cases of revision THA. After receiving the results of the bacterial culture, we prescribed an additional course of oral antibiotics to the patient. This included intravenous cefazolin for 2 days and an additional course of oral antibiotics (minocycline + sulfamethoxazole-trimethoprim) for 17 months as prophylactic treatment. The CRP level normalized at 4-months postsurgery and the white blood cell count normalized within 6 months after the revision surgery. The Harris Hip Score (HHS) improved from 29 before surgery to 86 postoperatively. However, the patient reported recurrent left hip pain 1.5 years after the second THA, with an elevated CRP level (range, 0.7-2.6 mg/dL). We planned a second revision surgery (third THA), via a posterior approach, to remove the remaining HO from the posterior hip joint (Figure 4(a)) and replace the femoral stem to control the infection. We planned to use a two-stage approach; however, the patient did not consent to it. Owing to the severe limitation of hip ROM, we could not expand the surgical site intraoperatively. Thus, we excised the scar tissue, especially around the femur, and removed existing osteophytes and excessive cement around the proximal site of the stem to allow the removal of the well-fixed stem. However, the preexisting cement could not be entirely removed. We used vancomycin-mixed cement and replaced the acetabular cup and femoral stem simultaneously via a single-stage exchange, considering the mild nature of the infection and the patient's preferences (Figure 4(b)). A repeat bacterial culture of fluid from the hip joint revealed *S. caprae* again, and the patient's HHS again improved from 64 preoperatively to 74 postoperatively.

According to our care guidelines, antibiotics should be administered intravenously following THA, with consideration of additional oral antibiotics for patients with a PJI. If inflammatory markers are elevated during the postoperative oral antibiotic course, lifelong suppressive oral antibiotic treatment should be considered. Based on these clinical practice guidelines, we prescribed oral antibiotics (minocycline + sulfamethoxazole-trimethoprim) to the patient for 3 months after the third THA. At the latest follow-up, the patient did not report any left hip pain, and the CRP level has remained within the normal range for >3 years. Further, plain radiographs did not reveal evidence of worsening HO (Figure 4(c)) compared with that on the radiograph obtained immediately after the third THA (Figure 4(b)).

## 3. Discussion

HO is a known complication of THA. The incidence of severe HO (Brooker grades III-IV) is 0.9–63% [16, 17]. The extreme severity of HO in our patient, which surrounded the entire hip joint, was considered unusual. Our patient had several of the recognized risk factors for HO after THA, including male sex, advanced age, use of a cemented prosthesis, previous surgery via anterolateral/direct lateral approaches, and PJI after primary THA. Similarly, we speculated that the patient's diabetes mellitus increased his susceptibility to CNS infection, which mediated the development of severe HO.

*S. caprae* is a commensal CNS strain that is primarily isolated from goat milk and located in the skin and mammary glands of goats. Some reports have suggested an association between *S. caprae* infection and PJI. Seng et al. [10] reported that 96% of PJIs caused by *S. caprae* were localized to the lower limbs, with 88% of these cases involving an orthopedic device and the risk being highest for joint prostheses (60%). *S. caprae* is not highly infectious; therefore, these infections tend to be mild and persistent. In the study conducted by Rodríguez Fernández et al. [15], only 8% and 23% of 13 cases of infection caused by *S. caprae* showed resistance to fluoroquinolones and penicillin, respectively. To date, there exists no established algorithm for antibiotic treatment. Nonetheless, in the case study by Rodríguez Fernández et al., patients were mainly treated using beta-lactams (46%) and fluoroquinolones (31%) [15]. To our knowledge, a relationship between severe HO and mild *S. caprae* has not been confirmed. Pommepuy et al. [18] reported two cases of bilateral single-stage revision THA in patients with an *S. caprae* infection. HO in one of these patients was severe, as in our case. However, Pommepuy et al. did not indicate a possible association between severe HO and mild infection.

We acknowledge the inherent limitation of identifying a causal relationship based on one case alone. However, because of the extreme severity of HO in our case due to a persistent *S. caprae* infection, we examined a possible relationship. Prolonged infection causes trauma within the surgical field, which can complicate the surgical procedure. By activating osteoinductive factors, which help differentiate the mesenchymal stem cells into osteoprogenitor cells [19], prolonged local inflammation can mediate the propagation of fibroblasts that undergo metaplastic transformation into fibrocartilage, resulting in HO [20].

Several treatments for HO are available, including the use of bisphosphonates, nonsteroidal anti-inflammatory drugs, radiation therapy, and operative interventions [1, 5]. In our case, we did not prescribe nonsteroidal anti-inflammatory drugs because of the patient's history of gastric ulcers. In addition, the patient did not consent to radiation therapy. The presence of extremely severe HO limited the surgical field, which necessitated two revision surgeries. Although the direct lateral approach is a known risk factor for HO, we performed the revision THA (second THA) through the direct lateral approach to achieve a sufficient anterior-to-posterior view of the surgical field. Despite this broad surgical approach, we could not control the infection, which required a second revision surgery (third THA) with further expansion of the surgical field to remove the remaining HO and replace the femoral stem. Although we acknowledge the possibility that the infection was controlled by the further expansion of the surgical field at the time of the first revision surgery (second THA) and the replacement of the femoral stem, the femoral stem was well-fixed, which may have enabled us to avoid another revision surgery. Similarly, removal of the loosened cemented cup and its replacement with a polyethylene liner and allograft impaction may have been easier with this approach than with the paradoxical technique. To date, there is no established algorithm for the management of HO; however, it seems clear that revision THA is the only treatment for HO. Cobb et al. [21] reported a statistically significant increase in hip ROM by symptomatic HO resection following THA, with 3.5-year follow-up. Wu et al. [22] reported a 28.6% recurrence if HO resection was performed within 6 months after surgeries for acetabular fractures and 36.4% if performed after 6 months; however, there were no cases of severe HO recurrence.

In summary, *S. caprae* is a rare cause of PJI, causing low-grade infections. *S. caprae* is possibly associated with severe HO. The management of HO in the presence of PJI is difficult and can complicate or negatively influence the probability of a successful treatment for PJI, particularly if implants are retained because of poor access. A dual approach to the hip can enable adequate clearance of HO.

## References

[1] Shehab D., Elgazzar A. H., Collier B. D. (2002). Heterotopic ossification. *Journal of Nuclear Medicine*.

[2] Winkler S., Springorum H. R., Vaitl T. (2006). Comparative clinical study of the prophylaxis of heterotopic ossifications after total hip arthroplasty using etoricoxib or diclofenac. *International Orthopaedics*.

[3] Rosteius T., Rausch V., Pätzholz S. (2016). Incidence and risk factors for heterotopic ossification following periprosthetic joint infection of the hip. *European Journal of Orthopaedic Surgery and Traumatology*.

[4] Manrique J., Alijanipour P., Heller S., Dove M., Parvizi J. (2018). Increased risk of heterotopic ossification following revision hip arthroplasty for periprosthetic joint infection. *Arch Bone Jt Surg*.

[5] Ranganathan K., Loder S., Agarwal S. (2015). Heterotopic ossification: basic-science principles and clinical correlates. *The Journal of Bone and Joint Surgery. American Volume*.

[6] Biz C., Pavan D., Frizziero A., Baban A., Iacobellis C. (2015). Heterotopic ossification following hip arthroplasty: a comparative radiographic study about its development with the use of three different kinds of implants. *Journal of Orthopaedic Surgery and Research*.

[7] Zhu Y., Zhang F., Chen W., Zhang Q., Liu S., Zhang Y. (2015). Incidence and risk factors for heterotopic ossification after total hip arthroplasty: a meta-analysis. *Archives of Orthopaedic and Trauma Surgery*.

[8] Goel A., Sharp D. J. (1991). Heterotopic bone formation after hip replacement. The influence of the type of osteoarthritis. *The Journal of Bone and Joint Surgery*.

[9] Hürlimann M., Schiapparelli F. F., Rotigliano N., Testa E., Amsler F., Hirschmann M. T. (2017). Influence of surgical approach on heterotopic ossification after total hip arthroplasty – is minimal invasive better? A case control study. *BMC Musculoskeletal Disorders*.

[10] Seng P., Barbe M., Pinelli P. O. (2014). *Staphylococcus caprae* bone and joint infections: a re-emerging infection?. *Clinical Microbiology and Infection*.

[11] d'Ersu J., Aubin G. G., Mercier P., Nicollet P., Bémer P., Corvec S. (2016). Characterization of Staphylococcus caprae clinical isolates involved in human bone and joint infections, compared with goat mastitis isolates. *Journal of Clinical Microbiology*.

[12] Shuttleworth R., Behme R. J., Mcnabb A., Colby W. D. (1997). Human isolates of Staphylococcus caprae: association with bone and joint infections. *Journal of Clinical Microbiology*.

[13] Allignet J., Galdbart J. O., Morvan A. (1999). Tracking adhesion factors in Staphylococcus caprae strains responsible for human bone infections following implantation of orthopaedic material. *Microbiology (Reading)*.

[14] Blanc V., Picaud J., Legros E. (1999). Infection after total hip replacement by Staphylococcus caprae. Case report and review of the literature. *Pathol Biol (Paris)*.

[15] Rodríguez Fernández L., Martín Guerra J. M., Dueñas Gutiérrez C. J. (2020). Role of Staphylococcus caprae in nosocomial infection. *Enfermedades Infecciosas y Microbiología Clínica*.

[16] Higo T., Mawatari M., Shigematsu M., Hotokebuchi T. (2006). The incidence of heterotopic ossification after cementless total hip arthroplasty. *The Journal of Arthroplasty*.

[17] Vasileiadis G. I., Amanatullah D. F., Crenshaw J. R., Taunton M. J., Kaufman K. R. (2015). Effect of heterotopic ossification on hip range of motion and clinical outcome. *The Journal of Arthroplasty*.

[18] Pommepuy T., Lons A., Benad K., Beltrand E., Senneville E., Migaud H. (2016). Bilateral one-stage revision of infected total hip arthroplasties: report of two cases and management of antibiotic therapy. *Case Reports in Orthopedics*.

[19] Board T. N., Karva A., Board R. E., Gambhir A. K., Porter M. L. (2007). The prophylaxis and treatment of heterotopic ossification following lower limb arthroplasty. *Journal of Bone and Joint Surgery. British Volume (London)*.

[20] Freeman T. A., Parvizi J., dela Valle C. J., Steinbeck M. J. (2010). Mast cells and hypoxia drive tissue metaplasia and heterotopic ossification in idiopathic arthrofibrosis after total knee arthroplasty. *Fibrogenesis & Tissue Repair*.

[21] Cobb T. K., Berry D. J., Wallrichs S. L., Ilstrup D. M., Morrey B. F. (1999). Functional outcome of excision of heterotopic ossification after total hip arthroplasty. *Clinical Orthopaedics and Related Research*.

[22] Wu X. B., Yang M. H., Zhu S. W. (2014). Surgical resection of severe heterotopic ossification after open reduction and internal fixation of acetabular fractures: a case series of 18 patients. *Injury*.

